# Weighted gene correlation network analysis reveals novel regulatory modules associated with recurrent early pregnancy loss

**DOI:** 10.1042/BSR20193938

**Published:** 2020-06-15

**Authors:** Xiaoxiao Li, Yuanqi He, Cuifang Hao, Xiaona Li, Xue Li

**Affiliations:** 1 Weihai Municipal Hospital, Cheeloo College of Medicine, Shandong University, Jinan 264200, China; 2 Department of Obstetrics and Gynecology, Weihai Municipal Hospital, Cheeloo College of Medicine, Shandong University, Weihai 264200, China; 3 Reproductive Medicine Centre, Yuhuangding Hospital of Yantai, Affiliated Hospital of Qingdao University, Yantai 264000, China; 4 Department of Neonatology, Weihai Municipal Hospital, Cheeloo College of Medicine, Shandong University, Weihai 264200, China

**Keywords:** Gene Expression, prognostic markers, Recurrent early pregnancy loss, WGCNA

## Abstract

At present, the etiology and pathogenesis of recurrent early pregnancy loss (REPL) are not completely clear. Therefore, identifying the underlying diagnostic and prognostic biomarkers of REPL can provide new ideas for the diagnosis and treatment of REPL. The chip data of REPL (GSE63901) were downloaded from the NCBI Gene Expression Omnibus (GEO) database. Weighted Gene Co-Expression Network Analysis (WGCNA) was used to construct a co-expression module for studying the relationship between gene modules and clinical features. In addition, functional analysis of hub genes in modules of interest was performed. A total of 23 co-expression modules were identified, two of which were most significantly associated with three clinical features. The MEbrown module was positively correlated with cyclin E level and the out-of-phase trait while the MEred module was positively correlated with the effect of progesterone. We identified 17 hub genes in the MEred module. The functional enrichment analysis indicated that such hub genes were mainly involved in pathways related to cellular defense response and natural killer (NK) cell-mediated cytotoxicity. In the MEbrown module, we identified 19 hub genes, which were mainly enriched in cell adhesion molecule production, regulation of cellular response to growth factor stimulus, epithelial cell proliferation, and transforming growth factor-β (TGF-β) signaling pathway. In addition, the hub genes were validated by using other datasets and three true hub genes were finally obtained, namely *DOCK2* for the MEred module, and *TRMT44* and *ERVMER34-1* for the MEbrown module. In conclusion, our results screened potential biomarkers that might contribute to the diagnosis and treatment of REPL.

## Introduction

Recurrent early pregnancy loss (REPL), defined as two or more consecutive pregnancy losses at <10 weeks of gestation, affects 5% of couples aimed at childbirth [1]. While fetal chromosomal abnormalities represent the major factor behind sporadic miscarriages, they account for a smaller fraction of miscarriage events in REPL couples [2,3]. Currently available diagnostic procedures allow to identify clinical conditions increasing their risk to pregnancy failure and to offer appropriate management options only in 50% of REPL couples [1,4]. The known risk factors for developing of REPL are maternal thrombophilic disorders or antiphospholipid syndrome, uterine malformations, maternal immunological and endocrine disturbances, parental-balanced chromosomal rearrangements [4–6]. So far, the unexplained REPL accounts for more than half of REPL cases and its etiology is still not established despite years of investigation. Early recognition of a potential risk to pregnancy loss and systematic monitoring has beneficial effect in increasing live birth rates in REPL couples. The preferred medical supportive care includes determination of human chorionic gonadotrophin (hCG) rising serum concentrations in early pregnancy and frequent ultrasound examinations [7]. However, only a limited number of potential predictive biomarkers of threatening miscarriage have been proposed. Nowadays, research on expression modules of REPL is limited, which restricts the understanding of critical genes associated with the occurrence and recurrence of the disease. Although many studies have identified some important genes and pathways that have made great advances in the diagnosis and treatment of REPL [8–10], however, the prognosis of REPL patients is still very poor. Therefore, it is urgent to develop new markers to assess their malignant potential and prognosis.

Weighted Gene Co-Expression Network Analysis (WGCNA) is a method for exploring the correlation between genes and a given feature, which is used to perform weighted correlation network analysis [11,12]. The unique advantage of WGCNA is the ability to convert the gene expression data into co-expression modules that reveal the gene networks and signaling pathways [13,14]. WGCNA has been widely used in a variety of tumor research including uveal melanoma [15,16], breast cancer [17,13], and adrenocortical carcinoma [18], which is very helpful in identifying the candidate biomarkers. Furthermore, WGCNA not only helps compare the processes of differentially expressed genes (DEGs), but helps to clarify the correlation between genes in the co-expression module. To the best of our knowledge, the application of WGCNA in the identification of biomarkers of REPL has not been reported so far.

In the present study, we used the expression data of REPL to construct a co-expression module and found a module of interest specific to the recurrence of the disease. In addition, we identified the hub genes in the modules of interest and performed functional enrichment analysis, which contributed to clarify the main functions of each gene in the module. These findings may contribute to assess the prognosis of REPL and provide new insights into the treatment of REPL.

## Materials and methods

### Data

Two normalized datasets of gene chips, GSE26787 and GSE63901, used in the present study were downloaded from the Gene Expression Omnibus (GEO) database (http://www.ncbi.nlm.nih.gov/geo/) in NCBI. The GSE63901, based on the GPL10558 platform is a dataset from the study of Kosova et al. [19] in 2015, including 37 women of European descent who have recurrent early pregnancy loss (REPL), defined as two or more records of unexplained miscarriage. Thirty-seven patients were classified into four groups: (1) patients with out-of-phase endometrial histology dating (*n*=10); (2) patients with abnormally elevated cyclin E levels (*n*=9); (3) patients with normal cyclin E levels and in-phase histology dating; (4) patients with abnormally high cyclin E levels and treated with progesterone (*n*=5). Two biopsy samples were collected from each patient in groups 1–3, while two biopsy samples were collected from each patient before and after progesterone treatment in group 4. The dataset contained a total of 84 samples. Patients’ information is summarized in Supplementary Table S1. The GSE26787 dataset contained five normal, five repeated implantation failures (IF), and five recurrent miscarriage (RM) samples and was based on the GPL570 platform [20]. The clinical information of patients included in the GSE26787 study was also summarized in the Supplementary Table S1.

### Weighted gene correlation network analysis and co-expression network construction

In this experiment, WGCNA, a typical system biology algorithm, was used to perform gene co-expression analysis on REPL [11]. First, using the hclustfunction to perform cluster analysis of the samples in the R WGCNA library, we detected and eliminated the outliers. After excluding outliers, clustering was performed according to gene expression levels in each sample to uncover the correlation between samples. The soft-thresholding power was determined according to the rule of the scale-free network, and the minimum power value at the plateau was taken as the parameter of the subsequent analysis, and the average connectivity of the gene under different Power values was counted. A gene clustering tree was constructed based on the correlation of intergene expression levels, and the co-expression module was identified by dynamic pruning method. The minimum number of genes in the module was set to 50. Modules with similar expression patterns were then merged according to the similarity of the module eigenvalues (0.75). The expression pattern of the module gene in each sample was shown by the module eigenvalue MEmagenta (the abscissa was the sample and the ordinate was the module), and the sample eigenvalue was used to draw the sample expression pattern heat map. The figure can be used to find out the modules that are significantly related to a particular clinical trait.

Genes or proteins do not work alone in the process of disease, but work together. There are several ways to describe the correlation between genes and genes. The degree of correlation between genes was calculated by WGCNA using the Topological overlap measure (TOM), which was more biologically significant [21,22]. The Pearson correlation test was used to verify the statistical significance of the relation between the module and clinical trait, and *P*<0.05 was considered to be statistically significant. For the studied clinical traits of REPL, we chose the module with the highest weighted correlation coefficient cor among all modules as the modules of interest for further analysis.

After screening out the interested modules, the weighted gene co-expression network was constructed using Cytoscape (v3.6.0) [23], and the hub genes were identified by the Molecular Complex Detection (MCODE) plugin. Each node in the network represents a gene, and the edges represent the regulatory relationship between genes. Gene regulatory network could help us accurately screen candidate genes that were potentially involved in the regulation of target genes, and could use the function of known genes to predict unknown gene function.

### Function annotation of the hub genes

Gene Ontology (GO) functional analysis and Kyoto Encyclopedia of Genes and Genomes (KEGG) pathway analysis were performed on the Hub genes using the R-package clusterProfiler software [24]. GO annotation results can be classified into three main bodies: Molecular function, Biological process, and Cellular component. After multiple test calibration, with false discovery rate (FDR) ≤ 0.05 was set as the threshold, GO term and Pathway satisfying this condition were defined as GO term and Pathway which were significantly enriched in module genes of interest and the Hub genes.

### The expression of hub genes in the interested modules

To further investigate the importance of these hub genes in the interested modules, we used the wilcox.test function of the R package [25] to detect the expression status of these genes. *P*<0.05 was considered to be statistically significant.

### Validation of hub genes

Differential gene expression analysis was performed on GSE26787 to detect genes differentially expressed between REPL and non-REPL samples to validate the hub genes. Differential expression analysis was performed using the edgeR package in R. Genes with log2FC (log of fold change) ≥2 and adjusted *P*-values <0.05 were considered as significant DEGs. Module hub genes were merged with the DEGs and the common genes were further receiver operator curve (ROC) analysis for final validation as true hub genes. The area under the curve (AUC) of the ROC was calculated to evaluate the diagnostic accuracy of the true hub genes. The AUC is the value of the Wilcoxon–Mann–Whitney statistic [26]. The caTools package in R was used for ROC analysis and AUC at 95% confidence interval (CI) was computed.

## Results

### Construction of co-expression modules of REPL

**Figure 1:**
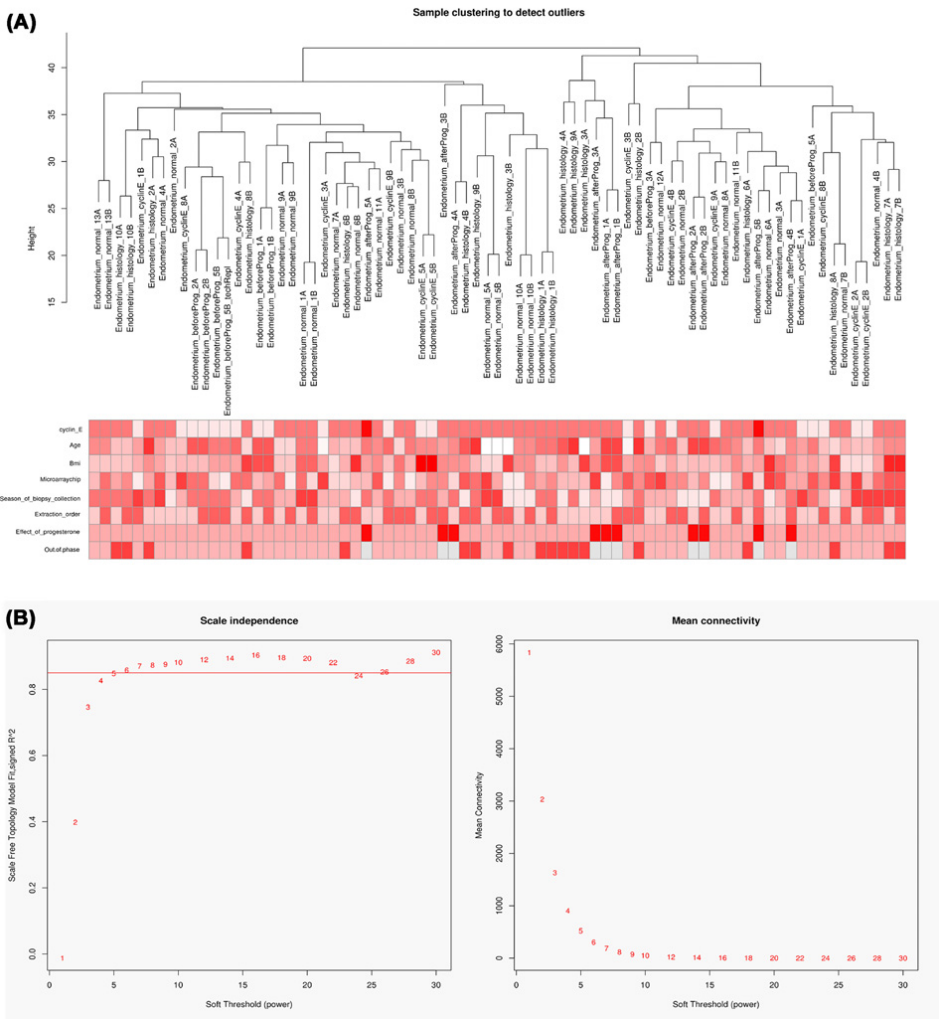
Sample dendrogram and soft-thresholding values estimation (**A**) Sample dendrogram and trait heatmap. (**B**) Scale independence and mean connectivity of various soft-thresholding values (β).

**Figure 2:**
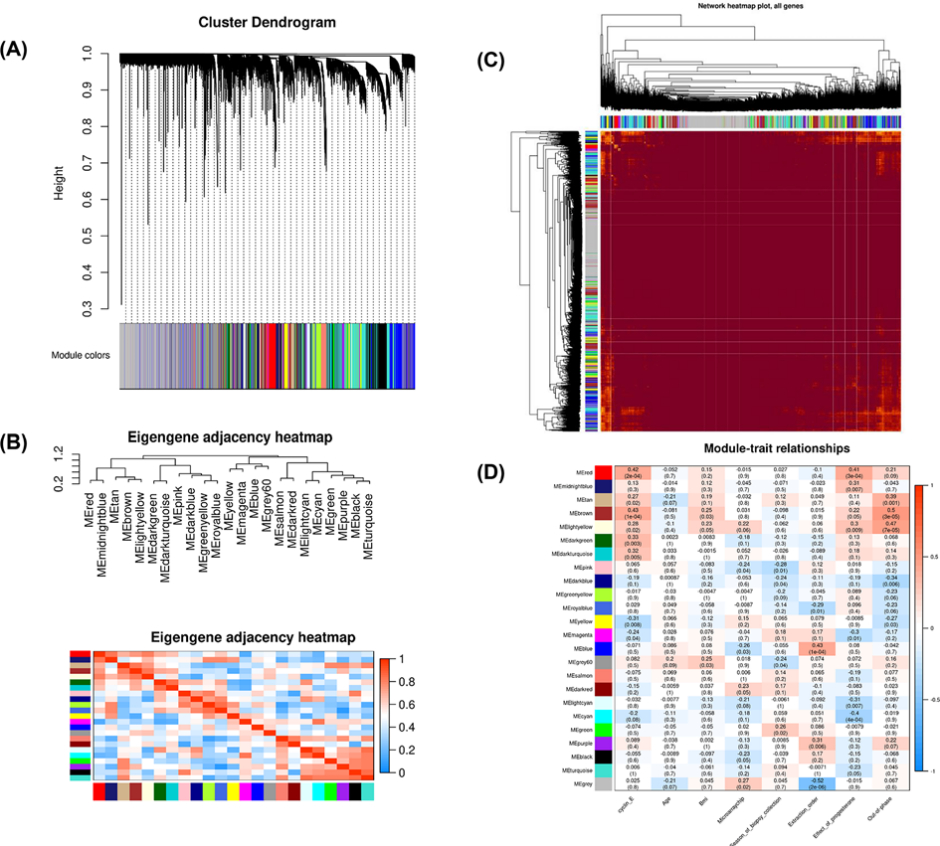
The module identification (**A**) Cluster dendrogram of all filtered genes enriched based on the dissimilarity measure and the cluster module colors. (**B**) Eigengene adjacency heatmap. (**C**) Network heatmap plot of all genes. (**D**) The module–trait relationships between the clinical traits and module eigengenes of REPL patients.

Subsequent to data processing, nine samples, namely GSM1560100, GSM1560101, GSM1560055, GSM1560056, GSM1560057, GSM1560058, GSM1560033, GSM1560034, and GSM1560086 were excluded and the expression matrix containing 15536 genes in 75 samples of REPL was used for WGCNA analysis. The Cluster tree displaying the relationship among the biological replicates of REPL samples was as shown in Figure 1A. The soft-threshold power is a key value used to power the correlation of the genes and affects the mean connectivity and the scale independence of co-expression modules. As shown in Figure 1B, the soft-threshold power was β = 6. At this power value, the scale independence was higher than 0.8 and the mean connectivity was higher. After generating the cluster dendrogram, 23 distinct gene co-expression modules, characterized by their unique module color, were identified in REPL (Figure 2A). The Eigengene adjacency heatmap depicting the interaction of the identified modules was reported in Figure 2B while the network heatmap plot of all of the genes was shown in Figure 2C. The heatmap depicting the correlation between the clinical traits and the co-expression modules was depicted in Figure 2D. From this heatmap, we observed that no co-expression module was significantly associated with the age of REPL patients. The modules significantly associated with the BMI were the MEbrown, MEgrey60, and MEpurple modules. The MElightgreen and the MEpurple co-expression modules were those influenced by the season of biopsy collection from the REPL patients whereas the extraction order was associated with the MEgreen, MEbrown and MEroyalblue co-expression modules. The modules significantly associated with the microarray chip were MEturquoise, MEdarkred, MEbrown, and MElightgreen co-expression modules. The modules influenced by the level of cyclin E were MEbrown, MEred, MElightcyan, MEdarkturquoise, MEmagenta, MEdarkgreen, MEpink, and MEgrey60 co-expression modules with MEbrown module being the most significant. The ‘out-of-phase’ trait was significantly associated with the MEmagenta, MEcyan, MEgrey60, MEbrown, and MEpink with the MEbrown as the most significant module. The effect of progesterone was significantly associated the MEbrown, MEred, MEmidnightblue, MEgrey60, MEdarkturquoise, MEtan, MElightyellow, and MEblue co-expression modules with the MEred module being the most significant module. For subsequent analysis, we chose the MEred module which was the most significantly associated with the effect of progesterone, and the MEbrown module due to the fact that it was significantly associated with the level of cyclin E, the effect of progesterone, and the ‘out-of-phase’ trait.

### Analysis of gene co-expression module associated with the effect of progesterone

**Figure 3:**
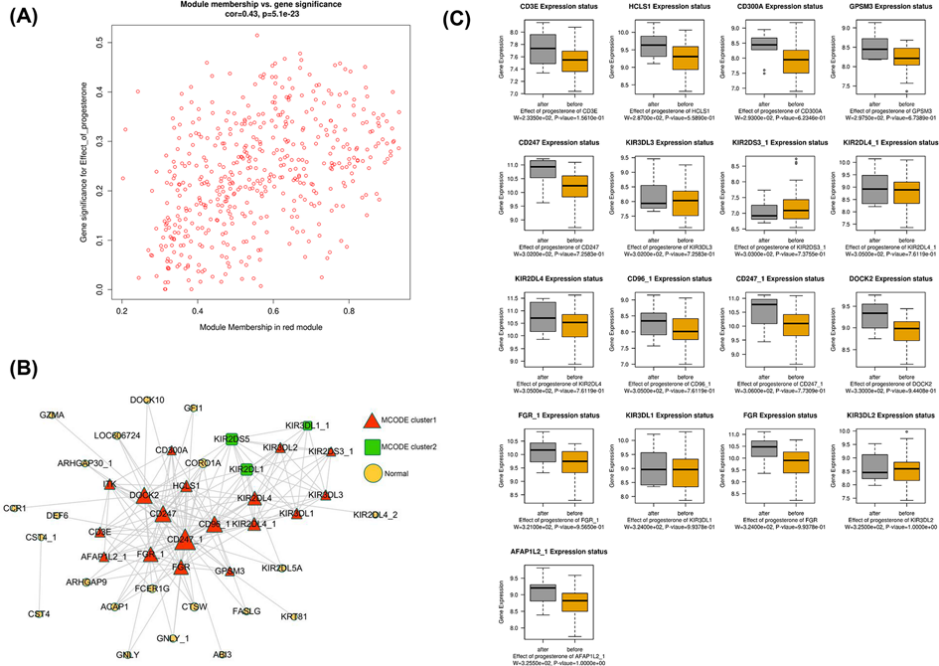
Analysis of gene co-expression module associated with the effect of progesterone (**A**) The scatter plot between the red module membership and the gene significance for effect of progesterone. (**B**) Construction of a co-expression network by WGCNA and screening for 18 hub genes. (**C**) The mRNA expression of 18 hub genes in the before or after treatment with progesterone.

**Figure 4:**
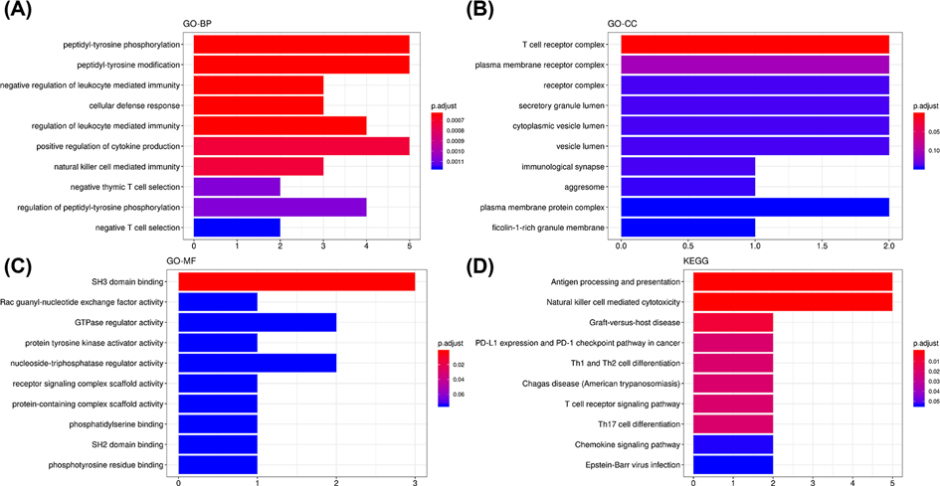
GO and KEGG pathway enrichment analysis of the MEred module genes (**A**) Biological process (BP) analysis. (**B**) Cellular component (CC) analysis. (**C**) Molecular function (MF) analysis. (**D**) KEGG pathway analysis.

As stated above, the MEred module was the most significantly associated with the effect of progesterone on patients with REPL. The MEred module was composed of 1275 genes. The correlation between the MEred module membership and the gene significance of the effect of progesterone is shown in Figure 3A. The module co-expression network constructed using the Cytoscape software was depicted in Figure 3B and showed strong interactions between the genes in this module. The MCODE Cytoscape plugin was used to extract the hub genes and the results indicated that two subnetworks could be extracted from the network. The first subnetwork contained 18 hub genes (nodes), namely CD300A, KIR3DL3, CD3E, KIR2DL4, HCLS1, DOCK2, CD247, KIR3DL2, CD96, GPSM3, FGR, KIR3DL1, KIR2DS3, FGR, CD247, AFAP1L2, and ITK. The boxplot of these genes was depicted in Figure 3C. It can be seen that nine of the hub genes (DOCK2, CD247, FGR, CD300A, CD247, HCLS1, GPSM3, FGR, and AFAP1L2) were significantly up-regulated following treatment with progesterone (Figure 3C). GO enrichment analysis was achieved on the genes in MEred module. The hub genes of this module were significantly enriched in the biological processes of peptidyl-tyrosine phosphorylation, peptidyl-tyrosine modification, negative regulation of leukocyte-mediated immunity, and cellular defense response (Figure 4A). The most enriched terms in cellular component were T-cell receptor complex, plasma membrane receptor complex, receptor complex, and secretory granule lumen (Figure 4B). The most representative term in molecular function was SH3 domain binding (Figure 4C). In KEGG enrichment analysis, we found that antigen processing and presentation, Natural killer (NK) cell-mediated cytotoxicity, Graft-versus-host disease, PD-L1 expression and PD-1 checkpoint pathway in cancer, and Th1 and Th2 cell differentiation were the most over-represented pathways (Figure 4D).

### Analysis of gene co-expression module associated with cyclin E level and out-of-phase trait

**Figure 5:**
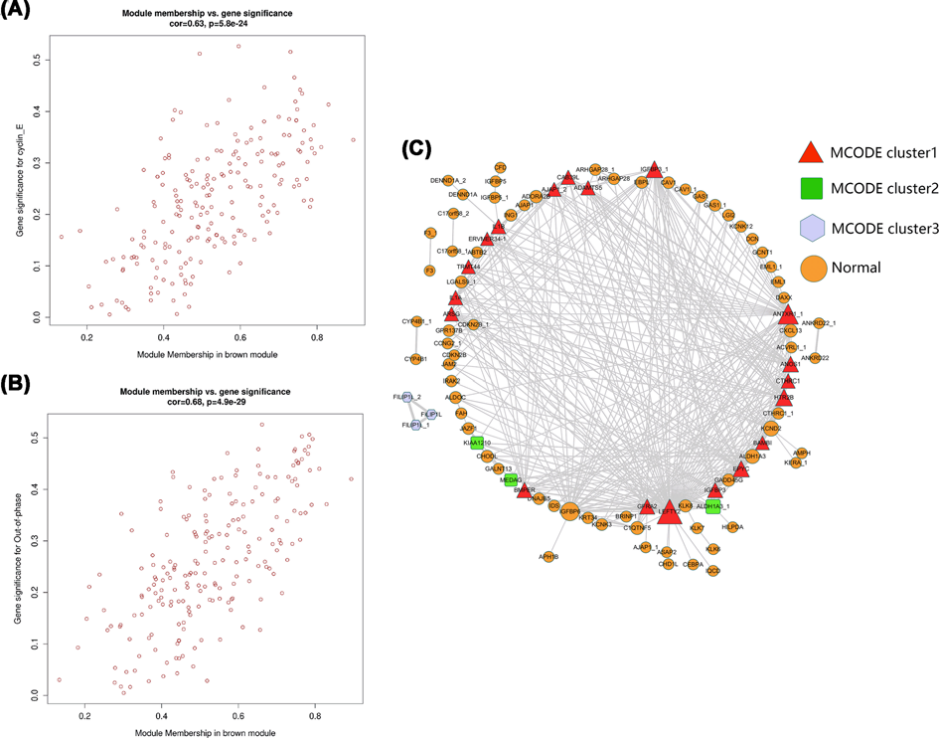
Analysis of gene co-expression module associated with the cyclin E level and out-of-phase trait The scatter plot between the MEbrown module membership and the gene significance for cyclin E level (**A**) and out-of-phase trait (**B**). (**C**) Construction of a co-expression network by WGCNA and screening for 19 hub genes.

**Figure 6:**
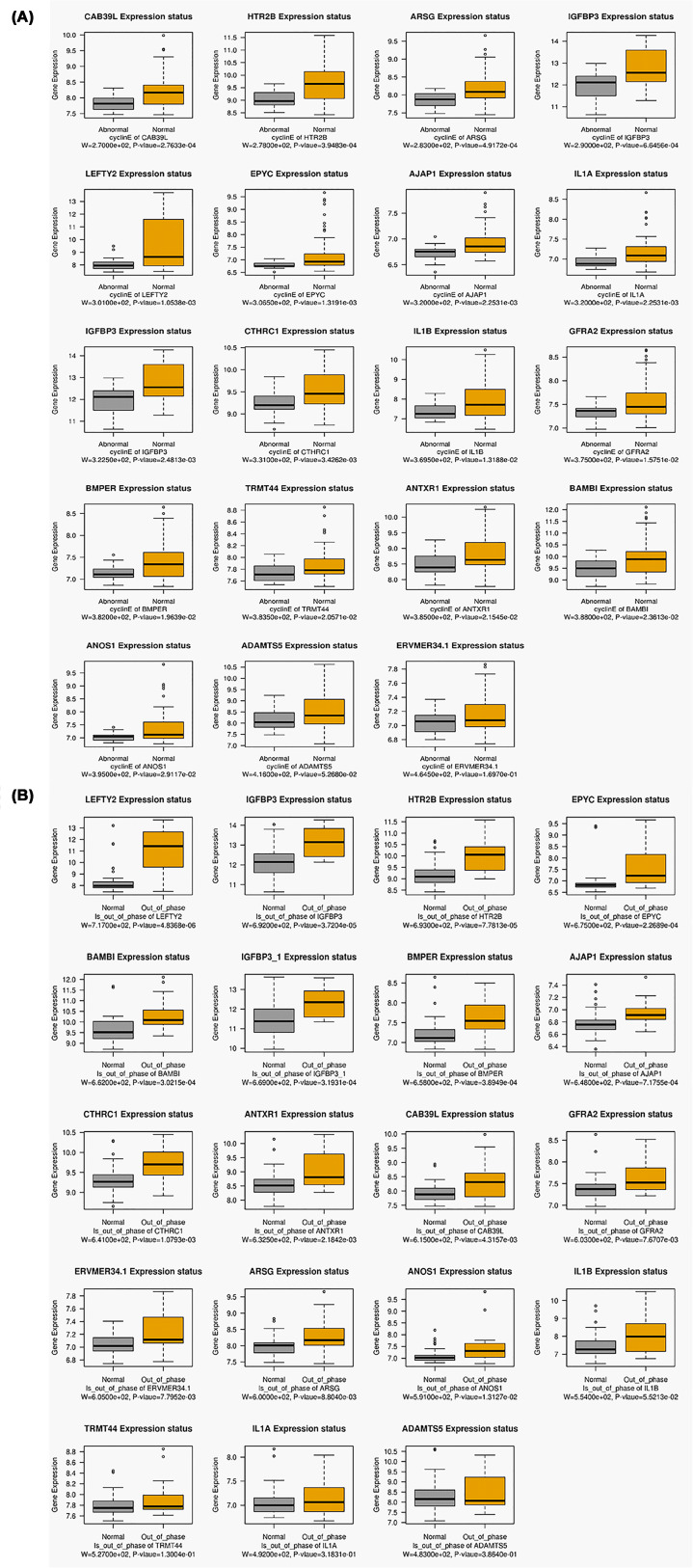
The mRNA expression of 19 hub genes in the MEbrown module (**A**) The expression of the 19 hub genes in REPL samples with normal and abnormal cyclin E levels. (**B**) The expression of the 19 hub genes in REPL samples with the out-of-phase and the normal groups.

**Figure 7:**
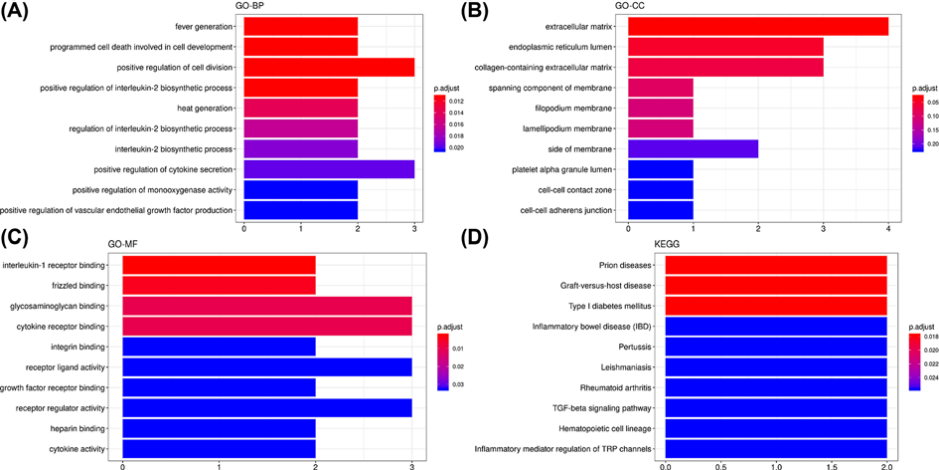
GO and KEGG pathway enrichment analysis of the MEbrown module genes (**A**) Biological process (BP) analysis. (**B**) Cellular component (CC) analysis. (**C**) Molecular function (MF) analysis. (**D**) KEGG pathway analysis.

As stated above, the MEbrown module was the most significantly associated with the cyclin E level and out-of-phase trait in REPL patients. The MEbrown module consisted of 204 genes. The correlations among the MEbrown module membership and the gene significance of the cyclin E level and out-of-phase trait are shown in Figure 5A,B. The module co-expression network was depicted in Figure 5C and showed strong interactions between the genes in this module. The MCODE extraction of the hub genes indicated a subnetwork of 19 hub genes (nodes), namely IL1B, IGFBP3, HTR2B, CTHRC1, left-right determination factor 2 (LEFTY2), EPYC, ADAMTS5, CAB39L, AJAP1, ANOS1, ERVMER34-1, IL1A, Anthrax toxin receptor 1 (ANTXR1), BMPER, ARSG, TRMT44, GFRA2, BAMBI, and IGFBP3 (Figure 5C). The boxplot of the expression of these genes was depicted in Figure 6A (for the cyclin E level) and Figure 6B (for the ‘out-of-phase’ trait). Except for ADAMTS5 and ERVMER34-1, there was a significant difference in the expression of the remaining genes between REPL samples with normal and abnormal cyclin E levels (Figure 6A). These DEGs were all down-regulated in abnormal cyclin E level group compared with the normal group (Figure 6A). Between out-of-phase and normal groups, four genes (IL1B, TRMT44, IL1A, and ADAMTS5) did not show significant difference while the remaining genes were all markedly up-regulated in the out-of-phase group (Figure 6B). GO enrichment analysis was achieved on the genes in MEbrown module. The hub genes of this module were significantly enriched in the biological processes of fever generation, programmed cell death involved in cell development, positive regulation of cell division, positive regulation of interleukin-2 biosynthetic process and heat generation among others (Figure 7A). The most enriched terms in cellular component were extracellular matrix, and endoplasmic reticulum lumen (Figure 7B). The most representative terms in molecular function were interleukin-1 receptor binding, frizzled binding, glycosaminoglycan binding, cytokine receptor binding, integrin binding, and receptor ligand activity (Figure 7C). In KEGG enrichment analysis, we found that prion diseases, graft-versus-host disease, type I diabetes mellitus, inflammatory bowel disease (IBD), pertussis, leishmaniasis, *Salmonella* infection, rheumatoid arthritis, transforming growth factor-β (TGF-β) signaling pathway, hematopoietic cell lineage, and inflammatory mediator regulation of TRP channels were the most over-represented pathways (Figure 7D).

### Differential genes expression analysis

**Figure 8:**
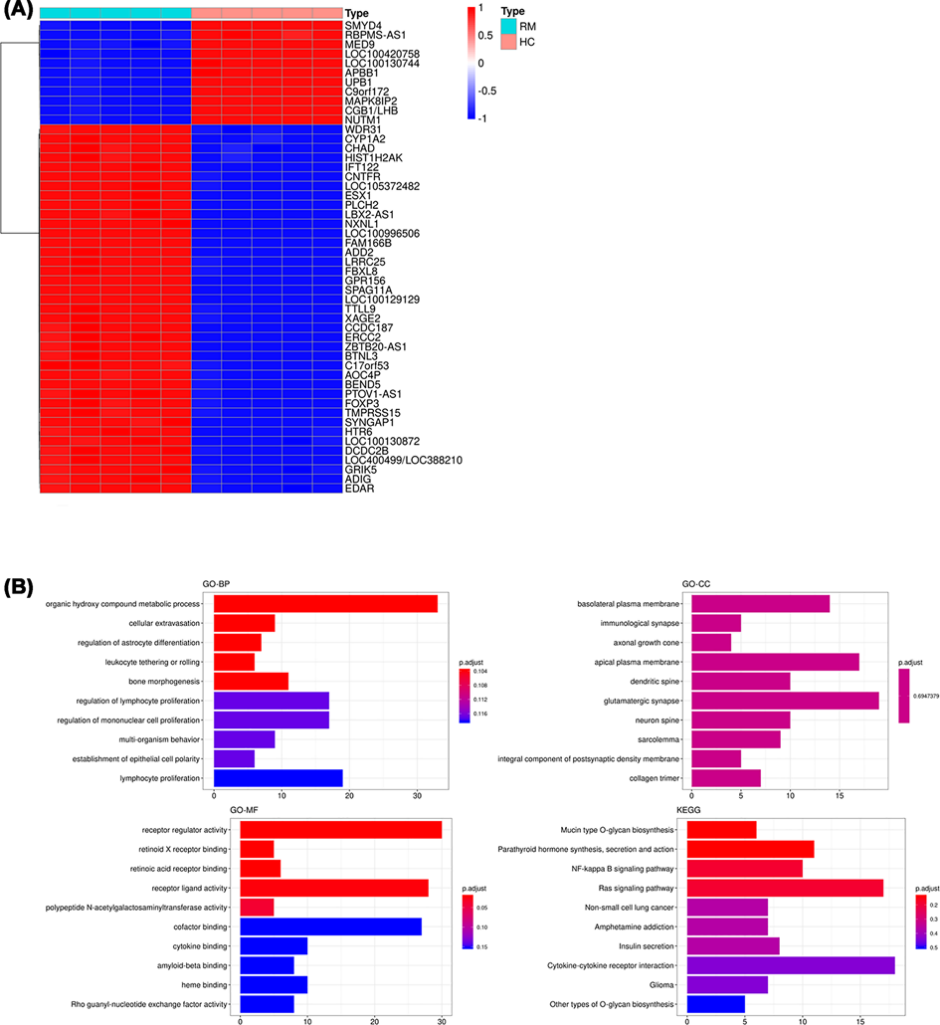
Differential expression analysis of genes between the REPL and non-REPL samples in GSE26787 (**A**) Heatmap showing the top 50 DEGs. (**B**) Functional enrichment analysis of DEGs.

To detect genes that were differentially expressed among REPL and non-REPL samples from GSE26787, differential expression analysis was performed. A total of 824 DEGs were identified and 600 of these DEGs were up-regulated in REPL. The top 50 DEGs were depicted in the heatmap, which included SMYD4, MED9, WDR31, and CYP1A2 (Figure 8A). Furthermore, functional enrichment analysis indicated that the DEGs were mainly enriched in organic hydroxyl compound metabolic process, cellular extravasation, receptor regulator activity, and Mucin type O-glycan biosynthesis pathway (Figure 8B).

### Validation of the hub genes

**Figure 9:**
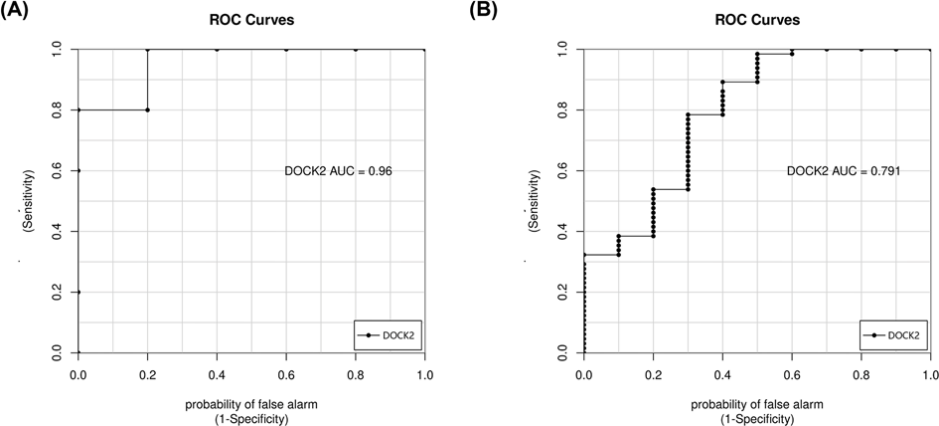
ROC curve analysis of the true hub genes associated with progesterone in two datasets (**A**) GSE26787 dataset; (**B**) GSE63901 dataset.

**Figure 10:**
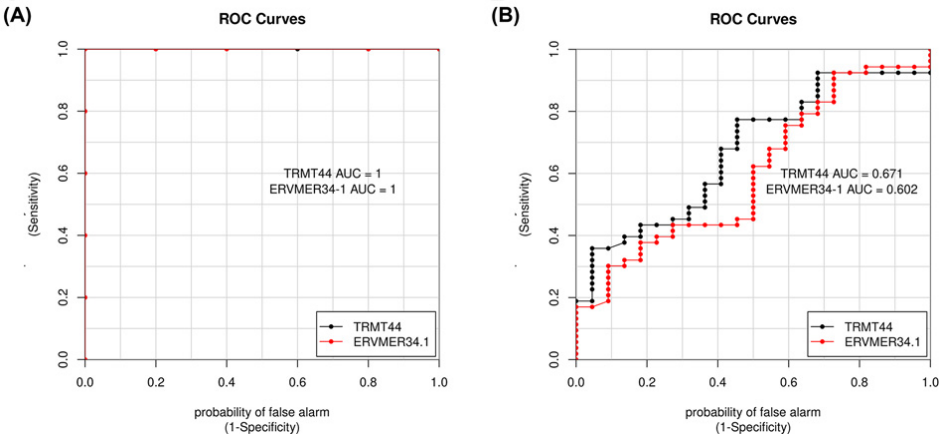
ROC curve analysis of the true hub genes associated with cyclin E levels and out-of-phase in two datasets (**A**) GSE26787 dataset; (**B**) GSE63901 dataset.

The intersection analysis of DEGs from GSE26787 and genes in the MEred module from GSE63901 allowed the identification of one common gene (DOCK2). Meanwhile, the intersection of DEGs from GSE26787 and genes in the MEbrown module from GSE63901 allowed the identification of two common genes (TRMT44, ERVMER34.1). To further test the value of the candidate true hub genes as prognostic biomarkers of REPL, ROC curves were performed and the AUCs (95% CIs) were calculated. As shown in Figure 9, the AUC of DOCK2 (the candidate true hub gene associated with progesterone) in GSE26787 was 0.96, while that in GSE63901 was 0.79. Additionally, as shown in Figure 10, the AUCs of TRMT44 and ERVMER34.1 (the candidate true hub genes associated with cyclin E and out-of-phase) in GSE26787 were both 1, while those in GSE63901 were respectively 0.67 and 0.60. These results suggested DOCK2, TRMT44, and ERVMER34-1 as potential biomarkers of REPL. Hence, these genes were regarded as the true hub genes associated with REPL.

## Discussion

In this work, WGCNA analysis was performed and 23 co-expression modules were generated based on 15536 genes from the 75 REPL samples. The aim of the present study was used elucidate the molecular pattern involved in REPL and the association of key genes with REPL features. The WGCNA approach was applied because of its advantages in uncovering co-expression modules and their correlation with sample traits, and its higher reliability and biological significance compared with other bioinformatics approaches. Our analysis allowed uncovering co-expression gene modules associated to important REPL traits such as the cyclin E level, the effect of progesterone, and the out-of-phase. The result of functional enrichment analysis indicated that there were significant differences in interactions among different modules, which was associated with their different functions in large part.

The MEred module which was significantly associated with the effect of progesterone was found to be mainly enriched in pathways related to cellular defense response, NK cell-mediated cytotoxicity, and antigen processing and presentation. All these pathways were related to immune response. Multiple studies have revealed that REPL is closely related to the dysregulation of immune response [27,28]. Our result suggested that the hub genes in the MEred module may regulate cellular defense response to affect the early embryo implantation. In addition, NK cells are a class of cytotoxic effector cells involved in the innate immune system that respond to a variety of cytokines such as type I interferon and interleukins, which help the host to produce immune responses [29]. In lymph nodes, NK cell-mediated killing of target cells affects T-cell responses, possibly by reducing antigen loading and/or target cell debris to promote antigen cross-presentation to CD8^+^ cytotoxic T cells [30]. A previous meta-analysis demonstrated significantly higher levels of NK cells (numbers and percentage) in REPL patients compared with healthy people [31]. We speculated that NK cells may function in cytotoxicity for endometrial cell to increase the risk of adverse pregnancy outcome. These results suggested that progesterone treatment could possibly affect immunity related pathways, which may be the cause of REPL pathogenesis. In addition, the MEbrown co-expression module was associated with cell adhesion molecule production, regulation of cellular response to growth factor stimulus, epithelial cell proliferation, and TGF-β signaling pathway. The TGF-β signaling has been shown to regulate cell growth, immune response, and inflammation [32]. Moreover, TGF-β is able to induce immature lymphocytes to maintain the homeostasis of the immune system [33]. This suggested that the genes influenced by cyclin E levels and the cycle phase are involved in cellular processes such as proliferation and adhesion and immature process; thus, the dysregulation of these genes leads to REPL. Therefore, we anticipated that MEbrown and MEred modules were the most important module in the pathogenesis of REPL. Furthermore, the MEpurple module was mainly enriched in tissue homeostasis and melanosome membrane and the MEgreen module was mainly enriched in cell projection that may corresponding to lead to the recurrence of REPL.

The two co-expression modules were constructed by the Cytoscape software and the hub gene were identified. Killer cell immunoglobulin-like receptors (KIRs) are the major inhibitory receptors of NK cells, and binding to MHC-I molecules can inhibit the killing of NK cells and protect normal cells from attack [34]. An allogeneic recognition system consisting of KIR and human leukocyte antigen C (HLA-C) can participate in human immune response under the regulation of NK cells [35]. A number of studies have shown that KIRs are significantly associated with transplant rejection, mainly related to their immune processes and associated with a variety of immune diseases, such as kidney transplantation [36,37], ankylosing spondylitis [38], and psoriatic [39,40]. Previous studies have shown that women with recurrent spontaneous abortion lack inhibitory KIRs (2DL1, 2DL2, and 2DL3) and cannot inhibit the killing activity of NK cells, leading to miscarriage [41]. In the present work many KIRs were detected as hub genes affected by the effect of progesterone in REPL patients. Another important class of genes was immune related genes such as CD96, CD247, and CD3E. The proteins encoded by these genes belong to the immunoglobulin superfamily, which may play important roles in the adhesion interaction between activated T and NK cells in the late stages of the immune response and also function in antigen presentation.

In the brown module, LEFTY2 and the ANTXR1 genes were the most relevant hub genes. LEFTY2 belongs to the TGF-β superfamily and is highly expressed by decidual stromal cells in the late stage of the menstrual cycle [42]. LEFTY2 has been shown to be involved in the progression of a variety of tumors including endometrial cancer [43]. Moreover, patients with ‘unexplained infertility’ have elevated LEFTY2 in the endometrium during the receiving period, indicating that abnormal expression of LEFTY2 results in infertility [44]. ANTXR1, also known as tumor endothelial marker 8 (TEM8), acts as a transmembrane receptor protein that activates its downstream signaling pathway by binding to anthrax toxin ligands, which in turn mediates its toxic portion into the cell [45]. Previous reports indicated that ANTXR1 is highly expressed in breast cancer tissues and is associated with prognosis [46,47].

In addition, our study identified a set of true hub genes with high relevance in REPL, of which three, namely DOCK2, TRMT44, and ERVMER34-1. DOCK2, a guanine-nucleotide-exchange factor, is predominantly expressed in hematopoietic cells and regulates the activation and migration of immune cells. Several studies revealed that DOCK2 is critical in the development of various inflammatory diseases and cancers [48–50]; however, its role in REPL is unknown. DOCK2 regulates cytotoxicity, degranulation, and IFN-γ secretion of NK cells [51]. Seshadri et al. have revealed that the levels of NK cells in REPL patients were significantly higher than those in healthy people [31], thus we speculated that DOCK2 plays significant role in the pathogenesis of REPL by regulating NK cells. A previous report indicated that TRMT44 plays a causal factor of partial epilepsy with pericentral spikes (PEPS) [52]. Thus, we inferred that TRMT44 may be associated with neurological troubles encountered in REPL, which needs further clarifications. ERVMER34-1 is encoding a full-length retroviral protein which could be detected in blood of pregnant women [53]. The study conducted by Heidmann et al. revealed that the expression of ERVMER34-1 is observed as early as at the eight-cell stage and persists at all of the subsequent embryonic stages [54], but the possible role of ERVMER34-1 in pregnancy still to be uncovered. Furthermore, the ROC analysis indicated that the identified three true hub genes are likely to be good biomarkers with high sensitivity and specificity for REPL prognosis. Thus, these true hub genes could open new avenues for the diagnostic and treatment of REPL.

However, there are some limitations in the present study. On one hand, small sample size limits the statistical power to identified the hub genes. On the other hand, further molecular biological experiments are needed to analyze and validate these hub genes to determine if they may be beneficial in the diagnosis or treatment of RM.

In conclusion, our study indicated that diverse co-expression gene modules are involved in REPL pathogenesis. We identified 17 hub genes which may be involved in the regulation of progesterone effect for REPL treatment, and 19 hub genes which played an important role in cyclin E level and out of phase for REPL development. Additionally, we validated the hub genes and finally obtain three true hub genes, namely DOCK2, TRMT44, and ERVMER34-1. These genes could serve as biomarkers for the diagnosis and treatment of REPL. The study opens new ways for the study of REPL and its understanding. Further studies are needed to validate useful genes in the panoply of genes found in the regulatory modules.

## Supplementary Material

Supplementary Table S1

## Abbreviations

ANTXR1: Anthrax toxin receptor 1
AUC: area under the curve
CI: confidence interval
DEG: differentially expressed gene
GO: Gene Ontology
IFN-γ: Interferon gamma
KEGG: Kyoto Encyclopedia of Genes and Genomes
KIR: killer cell immunoglobulin-like receptor
LEFTY2: left-right determination factor 2
MCODE: Molecular Complex Detection
NK: natural killer
PD-L1: programmed cell death-ligand 1
PD-1: programmed cell death protein 1
REPL: recurrent early pregnancy loss
ROC: receiver operator curve
RM: recurrent miscarriage
TGF-β: transforming growth factor-β
Th1: T helper 1
WGCNA: weighted gene co-expression network analysis
